# Evidence for the importance of post-transcriptional regulatory changes in ovarian cancer progression and the contribution of miRNAs

**DOI:** 10.1038/s41598-017-08502-z

**Published:** 2017-08-15

**Authors:** Mengnan Zhang, Lilya V. Matyunina, L. DeEtte Walker, Weixuan Chen, Haopeng Xiao, Benedict B. Benigno, Ronghu Wu, John F. McDonald

**Affiliations:** 1 Integrated Cancer Research Center, Georgia Institute of Technology, 315 Ferst Drive, Atlanta, GA 30332 USA; 2 School of Biological Sciences, and Georgia Institute of Technology, 315 Ferst Drive, Atlanta, GA 30332 USA; 3 Parker H. Petit Institute of Bioengineering and Bioscience, Georgia Institute of Technology, 315 Ferst Drive, Atlanta, GA 30332 USA; 4 Ovarian Cancer Institute, 960 Johnson Ferry Road, Suite 130, Atlanta, GA 30342 USA; 5 Department of Chemistry and Biochemistry, Georgia Institute of Technology, 950 Atlantic Drive, Atlanta, GA 30332 USA

## Abstract

High-throughput technologies have identified significant changes in patterns of mRNA expression over cancer development but the functional significance of these changes often rests upon the assumption that observed changes in levels of mRNA accurately reflect changes in levels of their encoded proteins. We systematically compared the expression of 4436 genes on the RNA and protein levels between discrete tumor samples collected from the ovary and from the omentum of the same OC patient. The overall correlation between global changes in levels of mRNA and their encoding proteins is low (r = 0.38). The majority of differences are on the protein level with no corresponding change on the mRNA level. Indirect and direct evidence indicates that a significant fraction of the differences may be mediated by microRNAs.

## Introduction

The last several decades have witnessed historic breakthroughs in the development of new high-throughput technologies to detect molecular changes associated with cancer onset and progression. The detection of these molecular changes, combined with appropriate computational methods, has proven extremely useful in the establishment of highly accurate diagnostic markers of the disease^1, 2^. The functional significance of the detected changes has proven more difficult to interpret because of our limited understanding of the underlying causal mechanisms involved^3^. A case in point is the relationship between changes in levels of RNA transcripts and corresponding changes (or lack thereof) in levels of their encoded proteins^4^. For example, high-throughput technologies have identified significant changes in patterns of mRNA expression between cancer patient primary and metastatic samples but the functional significance of these changes often rests upon the assumption that observed changes in levels of mRNA accurately reflect changes in levels of their encoded proteins^5, 6^. The validity of this assumption is far from confirmed and is, in fact, questionable in light of increasing evidence of the importance of post-transcriptional mechanisms in both the onset and progression of many cancers^7, 8^. We report here the results of a systematic comparison of the expression of 4436 genes on the RNA and protein levels between tumor samples collected from the ovary (OV) and omentum (OM) of same ovarian cancer (OC) patient. Consistent with other recent studies^9–11^, our results indicate that the overall correlation between differences in levels of mRNA and their encoding proteins is low (r = 0.38). We find that the majority of the differences in levels of expression are on the protein level with no corresponding change on the mRNA level implying the importance of post-transcriptional regulatory mechanisms. The results of gene ontology (GO) analyses further support this conclusion. Finally, we present evidence that a significant fraction of the discordant differences in levels of RNA and protein between the OV and OM cancer samples is mediated by microRNAs.

## Results

### The majority of changes in gene expression between tumor samples collected from the ovary and omentum of the same patient occur at the post-transcriptional level

To systematically explore the relationship between RNA and protein expression, we integrated quantitative transcriptional and proteomic profiles of cancer cells isolated by laser capture microdissection from bulk tumor samples collected from the ovary (OV) and the omentum (OM) of the same patient. Expression of mRNA was measured by microarray (Affymetrix Human Transcriptome Array 2.0) as previously described^6^. Protein expression was measured using a recently developed, highly sensitive mass spectrophotometric method^12^.

**Figure 1:**
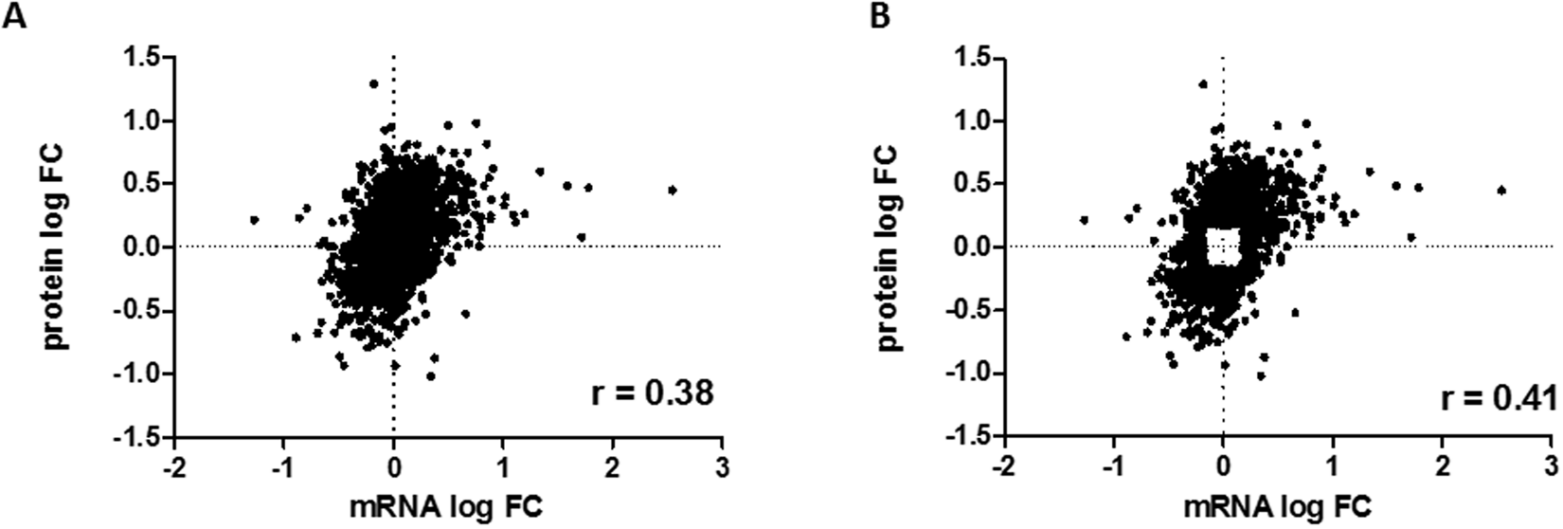
Correlation between changes in RNA and protein. Scatterplots with associated correlation coefficients(r) for (**A**) genes detected in both our mRNA and protein expression datasets (n = 4436); (**B**) genes displaying a significant (p = 3.376 × 10–79) change in expression between the OV and OM samples (n = 1946).

**Figure 2:**
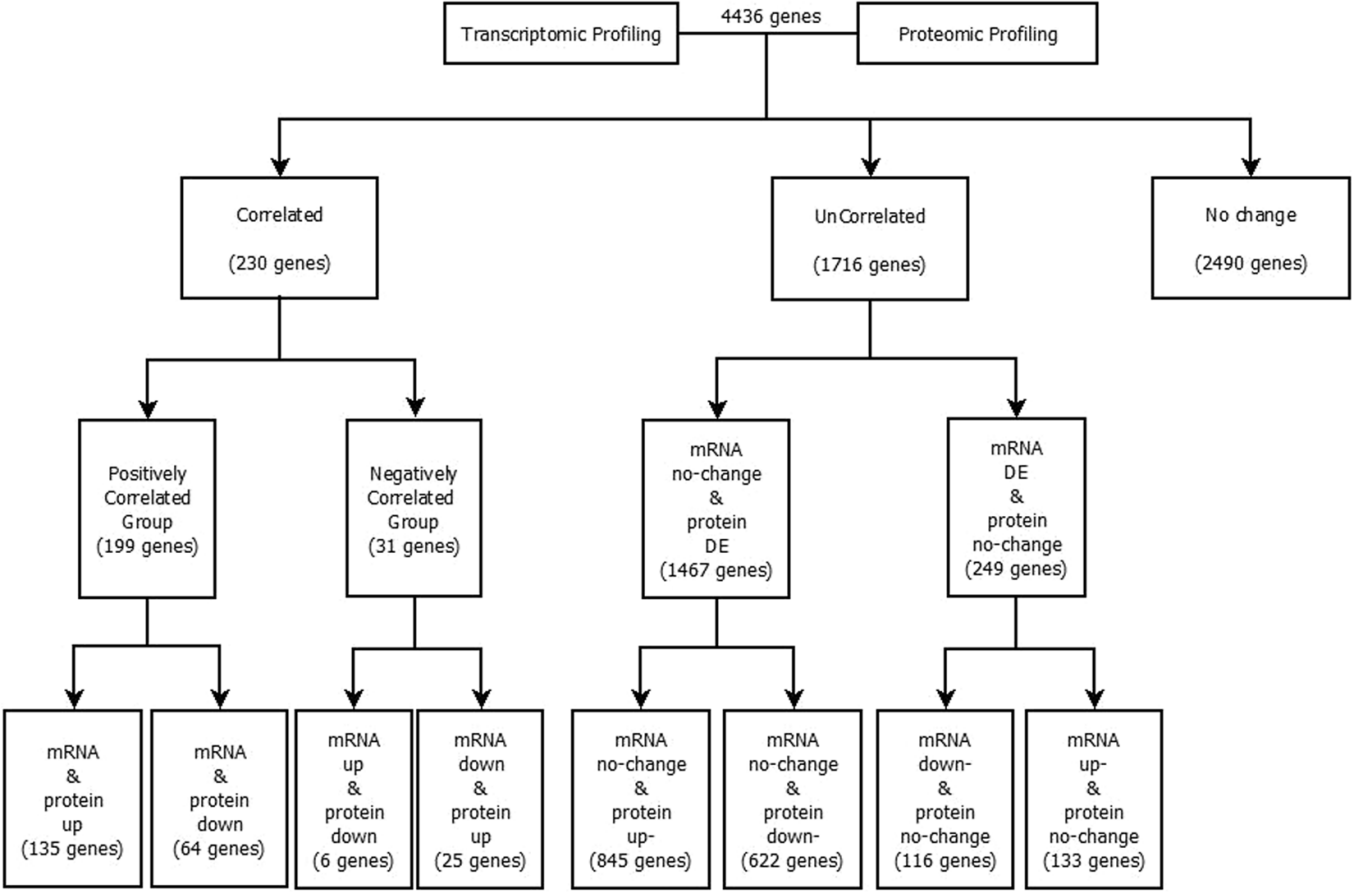
Diagram representing the classification of the integrated transcriptomic and proteomic datasets. Step 1: Integration of transcriptomic and proteomic profiling based on gene symbol matching. Step 2: Classification of differentially expressed genes into groups based upon changes in their respective mRNA and protein levels in OV and OM samples.

Of the 18,643 genes displaying detectable levels of RNA (Supplementary Table 1), the expression of only 4436 were detectable on the protein level (FDR < 0.01; Supplementary Table 2). The overall correlation between changes in levels of mRNA and protein encoded by these 4436 genes between the OV and OM samples was low (r = 0.38) (Fig. 1A), in part, because the majority of the genes displayed little or no change in expression between the OV and OM samples. Of the 4436 genes detected in both our mRNA and protein expression datasets (Fig. 2; Supplementary Table 3), the majority (2490 genes) displayed no significant change in expression (<1.5 fold change) on either the mRNA or protein levels. Of the 1946 genes displaying a significant change (>1.5 fold) in expression between the OV and OM samples, 230 were significantly differentially expressed on both the mRNA and protein levels, 1467 were significantly differentially expressed on the protein level but not on the RNA level and 249 on the RNA level but not on the protein level. The overall correlation between changes in RNA and protein for the 1946 significantly differentially expressed genes (Fig. 1B; r = 0.41, p = 3.376 × 10^−79^) is in general agreement with similar comparative studies previously carried out on a variety of mammalian tissues, tumors and/or cell lines^9–11, 13–15^. This overall correlation is not greatly affected by increasing the stringency of the cut-off value used in the analysis (Supplementary Table 4).

**Figure 3:**
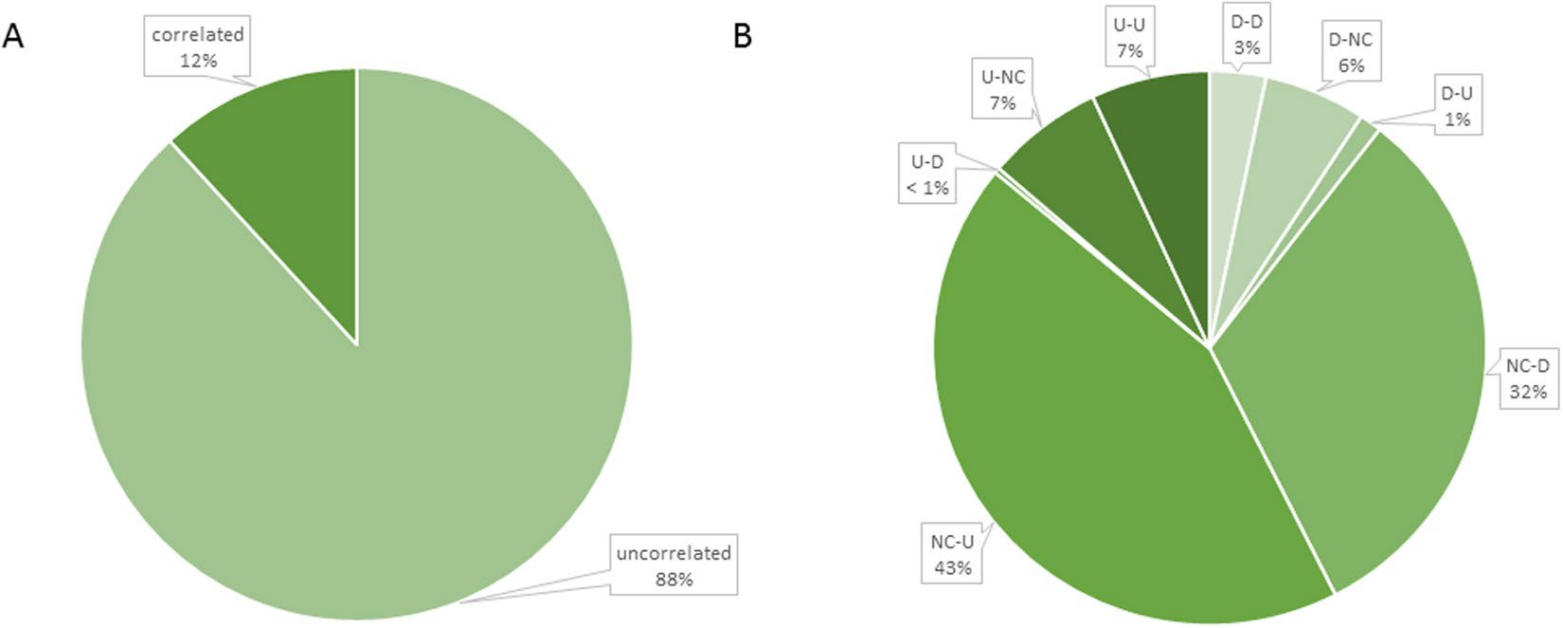
Pie chart showing the distribution of genes in correlated and uncorrelated groups. (**A**) The combined percentage of genes displaying correlated changes (U-U, D-D, D-U, and U-D) and uncorrelated changes (NC-D, NC-U, D-NC, and U-NC) in mRNA and protein levels between the OV and OM. (**B**) The percentage of genes in each subgroup of correlated and uncorrelated changes.

We further classified genes differentially expressed between the OV and OM samples into three groups based upon changes in their respective mRNA and protein levels: The *positively correlated* (*PC*) *group* is comprised of genes displaying positively correlated changes in mRNA and protein levels between the OV and OM samples [*i.e*. up (mRNA)-up (protein) (U-U); down-down (D-D)]; the *negatively correlated* (*NC*) *group* is comprised of genes displaying negatively correlated changes in mRNA and protein levels between the OV and OM samples [*i.e*., up (mRNA)-down (protein) (U-D); down-up (D-U)]; and the *uncorrelated* (*UC*) *group* is comprised of genes displaying significant changes in the expression of either protein or mRNA levels but not both [*i.e*., up (mRNA)-no change (protein) (U-NC); no change-up (NC-U); down-no change (D-NC); no change-down (NC-D)]. As shown in Fig. 3A, the combined percentage of genes displaying uncorrelated changes (NC-D, NC-U, D-NC, and U-NC) in mRNA and protein levels between the OV and OM samples (88%) far exceeds the percentage of genes (12%) displaying correlated changes. This suggests that the vast majority of changes in gene expression between the OV and OM samples involve processes occurring on the post-transcriptional/translational level. As shown in Fig. 3B, most of the uncorrelated changes are in the NC-U sub-group (43%) followed by the NC-D sub-group (32%), the U-NC (7%) and D-NC (6%) sub-groups. Most of the positively correlated changes are in the U-U subgroup (135/199 = 68%) with only 32% (64/199) of the positively correlated changes being in the D-D subgroup. The relatively few changes (1.3%) comprising the negatively correlated group are contained predominantly in the D-U subgroup (25 genes) with only six genes being in the U-D subgroup.

### Gene ontology analyses implicates EMT in the differences observed between samples and underscores the limitations of predictions drawn from RNA profiling alone

**Figure 4:**
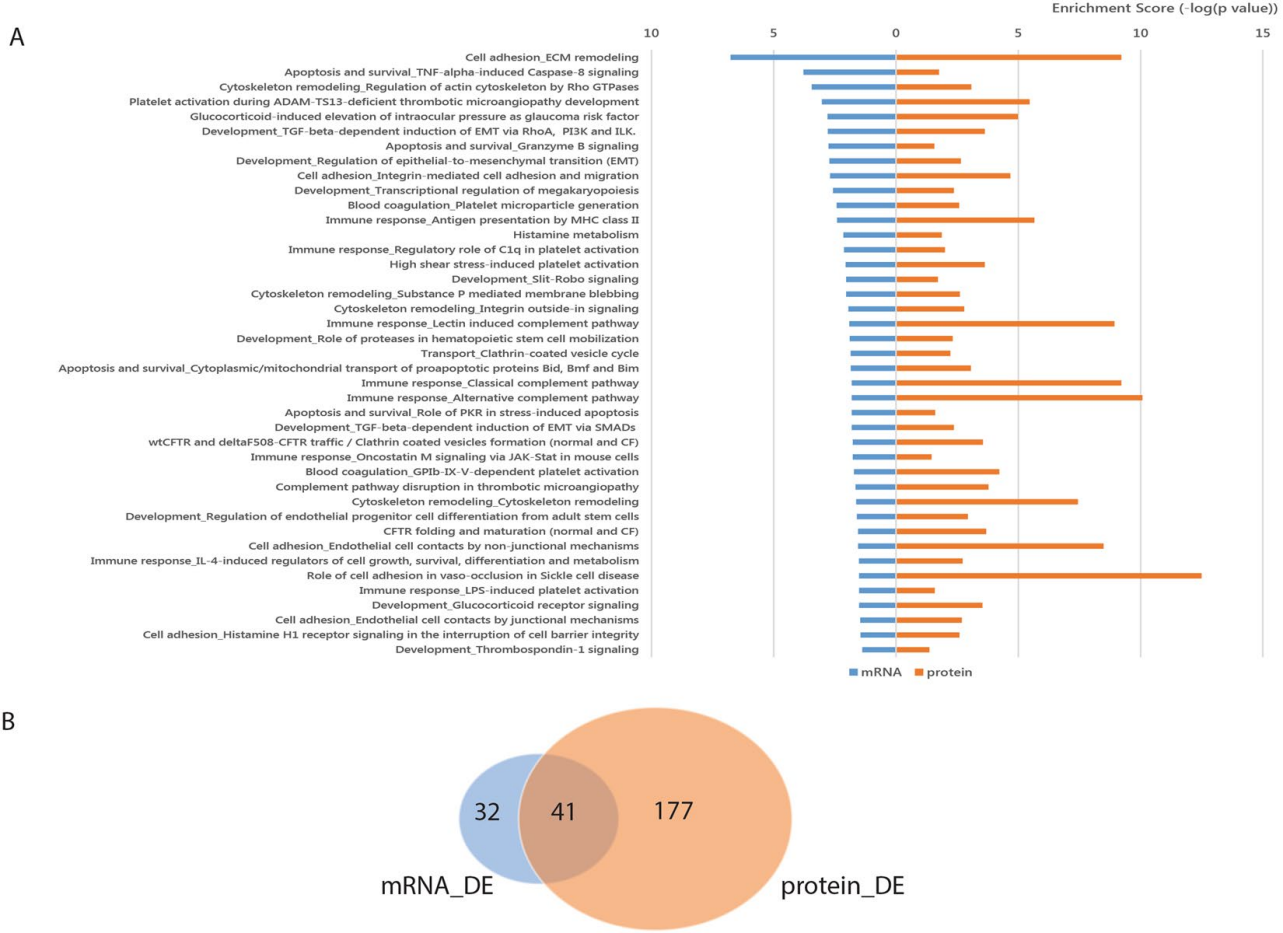
Results of GeneGo pathway enrichment analysis. (**A**) 41 GeneGo pathways significantly enriched in differentially expressed genes on either mRNA or protein level. (p-value < 0.05) (**B**) Venn diagram shows the number of enriched GeneGo pathways of each dataset that were found to be significantly enriched. (p-value < 0.05).

In an effort to evaluate the potential functional significance of the observed changes in RNA and protein expression between the OV and OM samples, we employed gene ontology analyses to compare biological pathways enriched for genes identified as significantly differentially expressed on the RNA and protein levels. Using the combined RNA and protein datasets (1946 genes), a total of 250 biological pathways were identified as being significantly (p < 0.05) enriched among genes differentially expressed between the OV and OM samples. Using the RNA expression dataset alone, 73 pathways were identified as being significantly enriched, while 218 were significantly enriched using the protein dataset alone (Supplementary Tables 5 and 6). There was an overlap of only 41 biological pathways (16%) enriched in the two datasets (Fig. 4A,B). More than half of the 41 overlapping biological pathways uncovered in our analysis (*e.g*., cell adhesion and cytoskeleton remodeling, *etc*.) are either directly or indirectly associated with epithelial-to-mesenchymal transition (EMT)- a cellular function believed to be critical in cancer metastasis^16, 17^.

While the 41 overlapping pathways constitute the majority of those predicted from the RNA dataset alone (41/73 = 56%), nearly half of the pathways predicted to be significantly overrepresented from the RNA dataset are likely spurious since these RNA changes are not manifest on the protein level. This finding coupled with the fact that >80% (177/218) of the pathways predicted from the protein dataset were not predicted from the RNA dataset points to the limitations of functional pathway predictions drawn from RNA profiling alone.

The fact that many pathways known to be central to EMT and other aspects of metastasis were significantly enriched among genes differentially expressed on the protein but not the RNA level (*e.g*., development-TGF-beta-dependent induction of EMT via MAPK, cell adhesion-role of tetraspanins in the integrin-mediated cell adhesion, cytoskeleton remodeling-Fibronectin-binding integrins in cell motility, *etc*.) further supports the functional importance of post-transcriptional/translational regulatory controls and underscores the importance of understanding the molecular mechanisms involved.

### Differences in microRNA (miRNA) expression contribute to post-transcriptional/translational changes between the OV and OM samples

The discordance between changes in levels of protein and their encoding mRNAs can be explained in a variety of ways including differences in relative rates of *in vivo* synthesis and stability^18^. While some differences in RNA/protein stability can be attributed to inherent differences in molecular structure, emerging evidence suggests that relative rates of both protein synthesis and RNA/protein stability are often post-transcriptionally regulated by microRNAs (miRNAs)^19^.

MiRNAs are small, non-encoding regulatory RNAs that can post-transcriptionally regulate levels of RNA and protein by degrading targeted mRNAs and/or by repressing translation of targeted mRNA transcripts^20^. To explore the possible role of miRNAs in the observed discordance between mRNA and protein levels between the OV and OM samples, we measured changes in levels of miRNAs and correlated these changes with corresponding changes in levels of mRNA and protein of their targeted genes. Differences in levels of miRNAs between the OV and OM samples were determined by microarray (Affymetrix GeneChip® miRNA 3.0 Array) and the mRNAs targeted by the differentially expressed miRNAs predicted using the miRanda-mirSVR algorithm^21^. Our focus was on genes displaying significant decreases in protein levels in the OM sample with no corresponding change in levels of mRNA (NC-D group). Our goal was to test the hypothesis that at least some of the observed discordance might be explained by the up-regulation of regulatory miRNAs.

The NC-D group is comprised of 622 genes, 592 of which are predicted to be targeted by 1100 miRNAs (Supplementary Table 7). Of these 1100 miRNAs, 101 are significantly differentially expressed between the OV and OM samples (Supplementary Table 8) and 48 of these are significantly up regulated in the OM sample (Supplementary Table 9). For example, the 10 most significantly up-regulated of these miRNAs and the number of their predicted gene targets in the NC-D and D-D groups is shown in Table 1. Interestingly, the gene targets of miRNAs significantly up regulated in the OM sample are contained in both the D-D and NC-D groups (Supplementary Tables 10 and 11). This implies that individual miRNAs up regulated in the OM sample may be regulating some genes on the transcriptional level (effecting down regulation of mRNAs and correlated changes on the protein level) and some genes on the translational level (effecting levels of protein with no correlated change on the mRNA level).

**Table 1:** The 10 most significantly up-regulated miRNAs in the OM sample and the number of their predicted gene targets in the NC-D and D-D groups.

miRNA	miRNA accession number	miRNA targets	
		NC-D group	D-D group
hsa-miR-139-5p	MIMAT0000250	150	17
hsa-miR-143*	MIMAT0004599	87	6
hsa-miR-150	MIMAT0000451	114	12
hsa-miR-196a	MIMAT0000226	66	5
hsa-miR-204	MIMAT0000265	154	11
hsa-miR-363	MIMAT0000707	131	7
hsa-miR-451	MIMAT0001631	32	0
hsa-miR-486-5p	MIMAT0002177	66	6
hsa-miR-675	MIMAT0004284	22	1
hsa-miR-675*	MIMAT0006790	60	3

**Figure 5:**
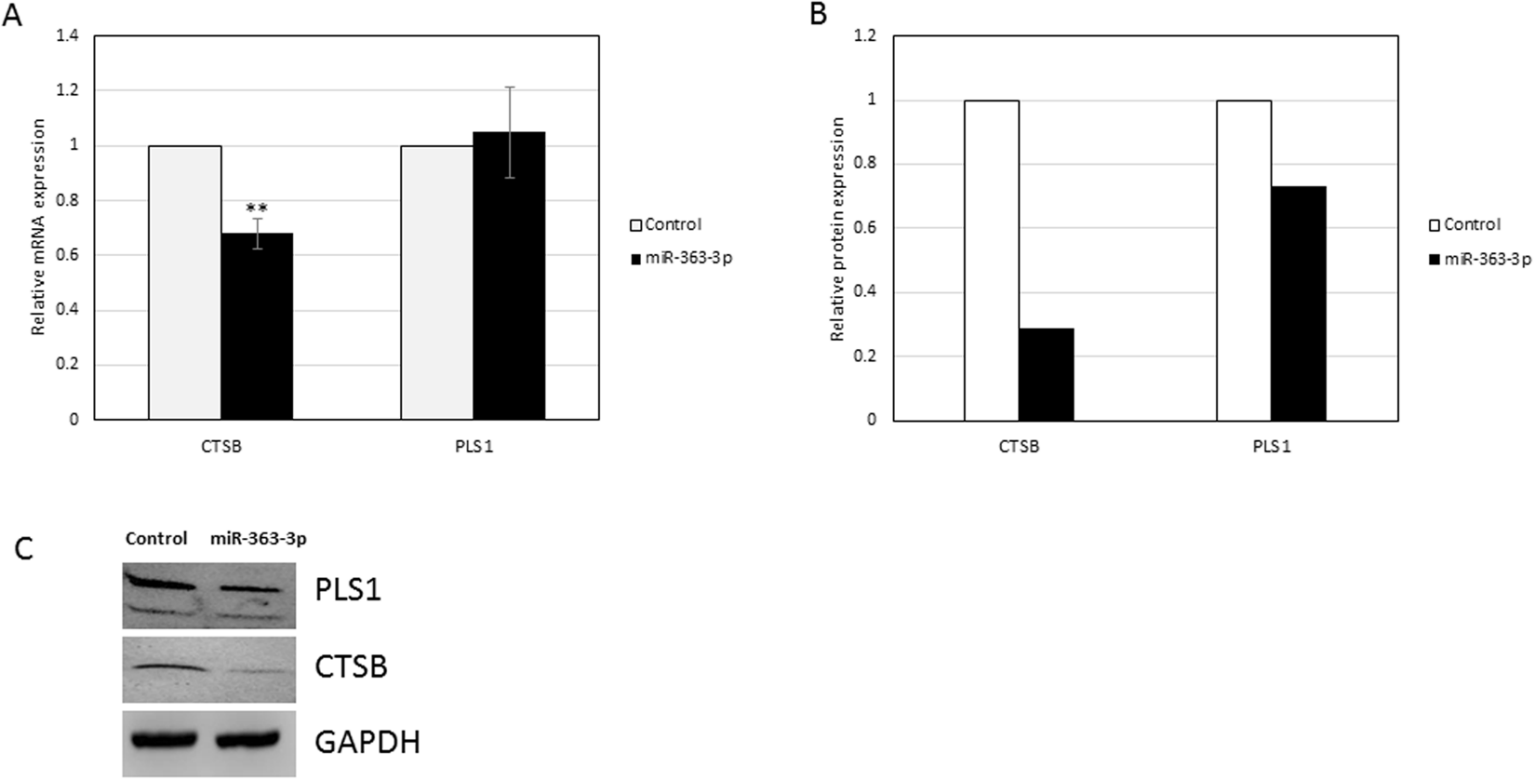
Effects of over-expression of miR-363-3p in HEY cells on the mRNA and protein expression of its predicted targets CTSB and PLS1. (**A**) Relative protein expression levels of CTSB and PLS1 as determined by Western blot. (**B**) Relative mRNA expression of CTSB and PLS1 as determined by qRT-PCR shows a significant decrease in CTSB mRNA levels but no significant changes on levels of PLS1. Expression values are normalized to negative control group and represent mean ± SD of at least three biological replicates each performed in three technical replicates. Asterisks represent statistically significant differences from the negative control group. (**p < 0.05) (**C**) Western blot analysis of CTSB and PLS1 proteins both display reduced levels of protein in the miR-363-3p group relative to negative control group.

In an effort to independently validate the observation that single miRNAs may preferentially regulate different genes on different levels (*i.e*., transciptionally vs. post-transcriptionally), we selected the most significantly up-regulated miRNA in OM, hsa-miR-363-3p, and exogenously over-expressed it in the well-characterized HEY ovarian cancer cell line^22^. Forty-eight hours after transfection of miR-363-3p in HEY cells, we extracted mRNA and protein, and monitored levels of two randomly selected genes, one from the D-D group (*CTSB*, Cathepsin B) and one from the NC-D group (*PLS1*, Plastin1). Western blots demonstrate that levels of CTSB and PLS1 protein are both decreased in cells in which hsa-miR-363-3p was over expressed (Fig. 5A,C). RT-PCR quantification of mRNA levels of these two genes showed a significant decrease in CTSB mRNA levels but no significant change in levels of PLS1 mRNA (Fig. 5B). The results of this experiment are consistent with the results of our RNA microarray and protein mass spectrometric analyses indicating that changes in levels of the same miRNAs may alternatively regulate levels of RNA and/or proteins in a gene-specific manner.

## Discussion

Modern DNA sequencing methods can identify genetic differences in cancer vs. normal tissues with nucleotide precision. Similarly, microarray, RNA sequencing and related high-throughput methodologies can quantitate changes in levels of gene expression on the RNA level with remarkable accuracy. The problem is that although the consequences of nearly all genome-wide molecular level changes manifest their functional significance on the protein level, global changes in the levels of protein are not easily and economically monitored by current methods. As a consequence, global changes in protein levels associated with cancer onset and progression are typically not directly measured but rather inferred from more easily monitored changes on the RNA level. The validity of these inferences is often questionable and, in some instance, may be misleading^7, 8^. For example, modern cancer medicine is rapidly embracing the molecular profiling of patient tumors in order to personalize targeted gene therapies^23^. Significant over-expression of a particular “cancer driver” gene on the RNA level may suggest treatment with a chemical inhibitor of the protein encoded by the over-expressed RNA. This may be a reasonable therapeutic strategy but only if elevated levels of mRNAs are an accurate reflection of levels of the targeted proteins.

The purpose of this study was to explore the system-wide relationship between differences in gene expression on the RNA and protein levels between tumor samples collected from the same patient. Using recently developed proteomic methodologies, we were able to explore this question on a global, level by directly comparing the expression of 4436 genes simultaneously on the RNA and protein levels.

We compared system-wide changes in levels of RNA and protein between discrete tumor samples collected from the ovary and the omentum of the same OC patient. Comparing molecular profiles between samples collected from the same patient removes the ambiguities associated with between-patient variation and allowed us to focus on changes associated with tumor progression within a single patient. Our results indicate that the majority of changes in mRNA and protein expression are not correlated with one another, consistent with emerging evidence for the importance of post-transcriptional/translational regulation in various aspects of cancer onset and progression^7^.

There remains controversy as to whether OC metastases originate from primary tumors in the fallopian tube, in the ovary or both^24^. While our studies do not resolve this controversy, our gene ontology (GO) analyses indicate that, for the patient analyzed in this study, the majority of biological pathways associated with the RNA and protein level changes between the OV and OM samples are associated with EMT. This is consistent with the growing body of evidence that EMT is critical to the metastatic process^16, 17^. The fact that the majority of changes in biological pathways between the OV and OM samples were predicted from the observed changes in protein and not RNA levels further supports the importance of post-transcriptional regulation in metastasis.

Because of the growing body of evidence implicating miRNAs in various aspects of cancer onset and progression^25^, we explored the possibility that miRNAs may be contributing to the observed discordance between changes in RNA and protein levels in our OV and OM samples. We measured changes in levels of miRNAs between OV and OM samples and correlated these changes with corresponding changes in levels of mRNA and protein of their targeted genes. Our focus was on testing the hypothesis that at least some of the genes displaying significant decreases in protein levels in the OM sample with no corresponding change in levels of mRNA (NC-D group) might be explained by the up regulation of regulatory miRNAs. Consistent with this hypothesis, we found that gene targets of miRNAs significantly up regulated in the OM sample are down regulated on the RNA and or protein levels. In some instances, individual miRNAs up regulated in the OM sample are associated with down regulation of their targeted mRNAs with a correlated down regulation on the protein level. In other instances, the same miRNAs up regulated in the OM sample were associated with significant down regulation of their targeted proteins but no correlated change in levels of their encoding mRNAs indicating a miRNA-mediated regulatory block on the translational level. This suggests that individual miRNAs up regulated in the OM sample may be regulating some genes on the RNA level and other genes on the protein level.

The possibility that a single miRNA may preferentially regulate different genes on different levels (*i.e*., transcriptional vs. translational) was corroborated by *in vitro* studies. Although the extensiveness and mechanisms underlying this phenomenon remains to be determined, previous studies have shown that the association of miRNAs with RNA binding proteins can significantly affect the levels (post-transcriptional and/or translational) on which individual miRNAs regulate their target genes^26^.

Collectively, our findings indicate that a significant fraction of the discordance in changes in RNA and protein levels between our samples are mediated by miRNAs and that miRNAs may contribute to the regulatory coordination of changes on the RNA and protein levels to enhance metastasis.

Overall, our findings are consistent with growing evidence of the importance of post-transcriptional/translational changes in the onset and progression of ovarian and other cancers and the potential significance of miRNAs in regulating the process.

## Methods

### Tissue collection

Cancer tissues from the right ovary and omental sites were collected from a woman with stage IIIc, grade 2/3 serous adenocarcinoma at Northside Hospital (Atlanta, GA) after informed consent was obtained under appropriate Georgia Institute of Technology Institutional Review Board protocols (H14337) according to previously described methods performed in accordance with the relevant guidelines and regulations^6^. Briefly, following resection, the tumor tissues were placed in cryotubes and immediately (<1 minute) frozen in liquid nitrogen. Samples were transported on dry ice to Georgia Institute of Technology (Atlanta, GA), and stored at −80 °C. After examination and verification by a pathologist, tissues were embedded in cryomatrix (Shandon, ThermoFisher, Waltham, MA). For each tissue sample, 8 μm frozen sections were cut and attached to uncharged microscope slides. Following dehydration and staining (HistoGene, LCM Frozen Section Staining Kit, Arcturus, ThermoFisher), slides were processed in an Autopix (Arcturus) instrument for laser capture microdissection (LCM). CapSure Macro-LCM Caps (Arcturus) were used to ensure purity of all collected cells. Approximately 30,000 cells were collected for each of the tissue samples.

### RNA extraction and amplification

RNA extraction and amplification were performed according to previously described methods^6^. miRNAs were isolated from the cells using the miRNeasy Micro KIT (Qiagen, Germantown, MD). The quality and quantity of miRNAs were assessed on the Bioanalyzer RNA Pico Chip (Agilent Technologies, Santa Clara, CA). Labeling of miRNAs was performed with the FlashTag Biotin HSR RNA Labeling Kit (Affymetrix, ThermoFisher) and hybridized to GeneChip® miRNA 3.0 Array chips (Affymetrix).

### Microarray analysis

Each individual RNA sample was analyzed both for miRNA and mRNA. miRNA profiling analysis was conducted on the GeneChip® miRNA 3.0 Array (Affymetrix). mRNA transcriptome analysis was analyzed using Gene Chip Human Transcriptome Array 2.0 (Affymetrix). In total, six miRNA and mRNA (two individual samples in triplicate) global expression data sets were generated in this study.

Raw miRNA and mRNA expression data were processed using Affymetrix Expression Console (EC) Software Version 1.4. Briefly, raw data probes were normalized using SST-RMA algorithm. The normalized expression values were log2 transformed. Differentially expressed mRNAs or miRNAs were identified through fold change and p-value calculated using two-tail Student t-test.

### Tissue homogenization, protein extraction and digestion

The minced tissue samples are dounced with tight dounce homogenizer in ice-cold homogenization buffer containing 0.25 M sucrose, 1 mM EDTA, 10 mM HEPES-NaOH, protease inhibitor mixture (Roche Diagnostics, Indianapolis, IN), pH 7.4 with 40 strokes on ice. The solutions were centrifuged at 1,000 g for 10 minutes at 4 °C. The supernatant was kept and the tissue pellets were re-suspended in RIPA buffer containing 100 mM 4-(2-hydroxyethyl)-1-piperazineethanesulfonic acid (HEPES), pH = 7.9, 150 mM NaCl, 0.5% sodium dodecyl sulfate (SDS), benzonase (1 U/mL), and protease inhibitor mixture (Roche Diagnostics). After complete solubilization of nuclei and digestion of genomic DNA, the lysate was centrifuged at 25,000 *g* for 10 minutes at 4 °C. The supernatants were combined and proteins were reduced by 5 mM DTT (56 °C, 25 min) and alkylated with 15 mM iodoacetamide (RT, 30 minutes in the dark). Proteins were purified with the chloroform-methanol precipitation method. Purified proteins were digested with Lys-C (the ratio of Lys-C and protein was about 1:50) at 31 °C for 15 hours followed by trypsin digestion at 37 °C for 4 hours. Digestion was quenched by the addition of 10% TFA to a final concentration of 0.4%, and the resulting peptides were purified using a Sep-Pak tC18 cartridge (Waters, Milford, MA).

### Peptide TMT labeling, fractionation and LC-MS/MS analysis

Purified and dried peptides from each sample were tagged with TMT reagents. Each sample was labeled using two channels (*i.e*. the peptides from the tumor tissue of the right ovary were labeled with channel 126 and 127 and the peptides of tumor tissue taken from the omentum were with 128 and 129). The four labeled peptide samples were combined and desalted using a tC18 Sep-Pak cartridge. Then peptides were fractionated using high-pH reversed phase high performance liquid chromatography (HPLC) (pH = 10). The sample was fractionated into 20 fractions. Each fraction was purified, dried and dissolved in a solvent containing 5% ACN and 4% formic acid (FA), and 4 μL was loaded onto a microcapillary column packed with C18 beads (Magic C18AQ, 5 μm, 200 Å, 100 μm × 16 cm) using a WPS-3000TPLRS autosampler (Dionex, Sunnyvale, CA). Peptides were separated by reversed-phase chromatography and detected in a hybrid dual-cell quadrupole linear ion trap – Orbitrap mass spectrometer (LTQ Orbitrap Elite, ThermoFisher) using a data-dependent Top 15 method. For each cycle, one full MS scan (resolution: 60,000) in the Orbitrap at 10^6^ AGC target was followed by up to 15 MS/MS for the most intense ions. Selected ions were excluded from further analysis for 90 s each. Ions with at least double charges were sequenced. MS/MS scans were activated by HCD at 40.0% normalized collision energy with 1.2 m/z isolation width, and detected in the orbitrap cell.

### Database searching, data filtering, and quantification

The raw files recorded by MS were converted into mzXML format. Precursors for MS/MS fragmentation were checked for incorrect monoisotopic peak assignments^27^. All MS/MS spectra were matched against a database encompassing sequences of all proteins in the Uniprot Human (*Homo sapiens*) database and common contaminants such as keratins using the SEQUEST algorithm (version 28)^28^. Each protein sequence was listed in both forward and reversed orientations to control and estimate the false discovery rate (FDR) of peptide identifications. The following parameters were used for the database search: 10 ppm precursor mass tolerance; 0.1 Da product ion mass tolerance; full trypsin digestion; up to two missed cleavages; variable modifications: oxidation of methionine (+15.9949); fixed modifications: carbamidomethylation of cysteine (+57.0214), N-terminus and lysine TMT modification (+229.1629).

The target-decoy method was employed to evaluate and further control FDRs of peptide identification^29, 30^, and linear discriminant analysis (LDA) was utilized to distinguish correct and incorrect peptide identifications based on multiple parameters such as XCorr, ΔCn, and precursor mass error^27, 31–33^. After scoring, peptides less than six amino acid residues were deleted and peptide spectral matches were filtered to a less than 1% FDR based on the number of decoy sequences in the final data set, then the data set was further filtered to <1% FDR at the protein level.

Quantification of confidently identified peptides was based on the TMT reporter ion intensities in MS^28^. The isotopic information provided by the company (ThermoFisher) was used to calibrate the measured intensities. The median intensity ratio for each unique peptide in each channel was obtained, and eventually the protein ratio is the median value of all unique peptides for the corresponding protein.

### Integration of transcriptomic and proteomic profiles

4436 genes detected by mass spectrometry (FDR < 0.01) were mapped to at least one probe set on the HTA 2.0 array by coding gene name matching. For genes with multiple mRNA probes corresponding to a single protein, the probe with the highest average expression level among OV and OM samples was used in the integrated dataset^34^.

### miRNA target prediction

The miRNA target prediction (based on mirSVR) was downloaded from microRNA.org (August 2010 release)^35^. The mirSVR score refers to targets of microRNAs with scores obtained from their support vector regression algorithm^21^. To reduce the occurrence of false positives, only predicted targets with a mirSVR score less than −0.1 were considered.

### Pathway enrichment analysis

Differentially expressed genes on mRNA and protein levels were employed for enrichment analysis using the MetaCore suite 6.29 build 68,613 (Thomson Reuters, New York, NY). Briefly, significantly perturbed pathways and process networks were identified by mapping differentially expressed genes onto manually curated GeneGO canonical pathway maps and cell process network models^36^. The statistical significance of enrichment was evaluated using p-values calculated based on hypergeometric distribution. Pathways were considered to be significantly enriched if their p-values were <0.05.

### Cell culture and microRNA transfection

The HEY cell line was kindly provided by Gordon Mills, Department of Molecular Therapeutics, University of Texas, MD Anderson Cancer Center. Cells were cultured in RPMI 1640 (Mediatech, Manassas, VA) supplemented with 10% FBS (Fetal Bovine Serum; Atlanta Biologicals, Lawrenceville, GA) and 1% antibiotic-antimycotic solution (Mediatech). For miRNA transfections, 6 × 10^4^ cells were seeded per well in 24-well plates. Cells at exponential phase of growth were transfected with 30 nM miRNA purchased as Pre-miR miRNA Precursors (Ambion, Austin, TX) using Lipofectamine 2000 (Invitrogen, Carlsbad, CA) and per the manufacturer’s instructions. Cells were allowed to grow for 48 hours before RNA isolation. Ambion Pre-miR miRNA Precursor Negative Control was used as a negative control.

### Real-time PCR

Total RNA was extracted from cells using the RNeasy Mini Kit (Qiagen). Four micrograms of RNA was reversed transcribed into cDNA using the Superscript III First-Strand Synthesis System (Life Technologies, ThermoFisher) according to the manufacturer’s instructions. Real-time PCR was performed using TaqMan® Real-Time PCR Master Mixes (Applied Biosystems, ThermoFisher) on a CFX96 Real-Time System (Bio-Rad, Hercules, CA). Expression values were normalized using *GAPDH* as a reference gene. Normalization and fold-change were calculated using the ∆∆C_t_ method.

### Western Blot

The total protein concentration of the supernatant was determined using a protein assay reagent kit (Bio-Rad). To the lysates, equal volumes of 2X Laemmli sample buffer were added and the samples were heated to 90 °C for 5 minutes. Equal amounts of proteins were separated by 4–20% gradient precast TGX gel (Bio-Rad) and transferred to nitrocellulose membrane (Bio-Rad). Membranes were blocked with 5% nonfat dry milk in 10 mM Tris buffered saline. After blocking, the membranes were probed with the primary antibody for overnight at 4 °C with gentle rocking. Antibodies used are against cathepsin B (CTSB antibody Cat # 365558, 1:100 dilutions; Santa Cruz Biotechnologies, Dallas, TX), I-Plastin (PLS1 antibody Cat # 386830, 1:200 dilutions; Santa Cruz Biotechnologies), and Glyceraldehyde-3-phosphate dehydrogenase (GAPDH antibody Cat # 47724, 1:100 dilutions; Santa Cruz Biotechnologies). Appropriate secondary antibodies were used at 1:5,000 dilutions (Santa Cruz Biotechnologies). After incubation with specific horseradish peroxidase–conjugated secondary antibody (goat anti-mouse horseradish peroxidase–(HRP) conjugated secondary antibody (sc-2005, Santa Cruz Biotechnologies), donkey anti-goat horseradish peroxidase–(HRP) conjugated secondary antibody (sc-2020, Santa Cruz Biotechnologies), protein was visualized using the enhanced chemiluminescence detection system (Pierce, ThermoFisher). The quantification of western blot bands was performed using ImageQuant software (GE Healthcare, Chicago, IL).

### Data availability

The microarray datasets supporting the conclusions of this article are available in the Gene Expression Omnibus (GEO) repository, (https://www.ncbi.nlm.nih.gov/geo/query/acc.cgi?acc = GSE100315). Other datasets supporting the conclusions of this article are included within the article and its Supplementary Files.

## Electronic supplementary material

Supplementary Table 1

Supplementary Table 2

Supplementary Table 3

Supplementary Table 4

Supplementary Table 5

Supplementary Table 6

Supplementary Table 7

Supplementary Table 8

Supplementary Table 9

Supplementary Table 10

Supplementary Table 11
